# Pharmacokinetics and Tissue Distribution of a Novel Bis-Chelated Gold(I) Diphosphine Compound, Bis(2,3-bis(tert-butylmethylphosphino)Quinoxaline)Aurate(I), in Rats

**DOI:** 10.3390/molecules24112082

**Published:** 2019-05-31

**Authors:** Ying Peng, Huanhuan Qi, Qingqing Chang, Yu Zhang, Weiyi Liu, Minyu Liu, Quanhai Liu, Guangji Wang, Jianguo Sun

**Affiliations:** 1 Key Lab of Drug Metabolism and Pharmacokinetics, State Key Laboratory of Natural Medicines, China Pharmaceutical University, 24 Tong Jia Xiang, Nanjing 210009, Jiangsu, China; 1020162518@cpu.edu.cn (Y.P.); you.qihuan@163.com (H.Q.); 15298377114@163.com (Q.C.); zhangyu0205com@sina.com (Y.Z.); 18609610906@163.com (W.L.); 2 Department of Pharmacology, Shanghai Institute of Pharmaceutical Industry, 1111 Zhong Shan Bei Yi Road, Shanghai 200437, China; liuminmyu_lmy@163.com (M.L.); liuquanhai_lqh@163.com (Q.L.)

**Keywords:** GC20, LC-MS/MS, pharmacokinetics, tissue distribution

## Abstract

GC20, a novel soluble bis-chelated gold(I)−diphosphine compound, has been reported as a promising anticancer candidate. Assessing the pharmacokinetic properties of GC20 is critical for its medicinal evaluation. First, a sensitive and specific liquid chromatography tandem mass spectrometry (LC-MS/MS) was developed and well validated to determine GC20 in rat plasma and rat tissue homogenate after one step protein precipitation. Chromatographic separation was achieved on an Angilent ZORBAX-C18 column (3.5 μm, 2.1 × 50 mm) with gradient elution and mass spectrometry was performed on a triple quadrupole in positive ion mode using an electrospray ionization source. This method was then applied to investigate the pharmacokinetics and tissue distribution of GC20 in rats after intravenous administration. The results showed that the plasma exposure of GC20 in vivo increased with increasing doses after a single dose. However, after multiple doses, a significant accumulation and a saturation at elimination were observed for GC20 in rats. Moreover, after intravenous administration, GC20 was widely distributed in various tissues, with the highest levels in the lung, spleen, liver, and pancreas, followed by the kidney and heart, while the lowest level was found in the brain. This is the first report on the pharmacokinetic properties of GC20.

## 1. Introduction

Metal-containing compounds such as platinum drugs have long been used clinically as first-line agents for cancer chemotherapy. Platinum drugs have developed rapidly, from one generation of cisplatin [1], two generations of carboplatin [2], nedaplatin [3], cycloplatin [4], to three generations of oxaliplatin [5], lobaplatin [6], all of which have shown high efficiency in both monotherapy and combination therapy. However, the side effects of these platinum drugs, the most significant systemic toxicity, limit their widespread use. In addition, long-term application of platinum drugs can easily lead to acquired drug resistance [7,8]. Nevertheless, the success of cisplatin has stimulated widespread interest in exploring compounds containing metals such as nickel, antimony, and gold as potential anti-cancer drugs with unique pharmacological, kinetic, and geometric properties [9].

Recently, gold complex has gained more and more attention in the treatment of cancer because of its strong anti-proliferation potency against cancer cells [10,11,12,13,14]. Among them, a novel bis-chelated gold(I) diphosphine complex, GC20(Bis(2,3-bis(tert-butylmethylphosphino)quinoxaline)aurate(I)), was identified as one most promising candidate for a new generation of gold(I)-based anticancer drugs [15]. It has been reported that GC20 has a strong inhibitory effect on the proliferation of a broad spectrum of cancer cell lines, including cisplatin-resistant ones. More importantly, GC20 has shown a remarkable selectivity between cancer cells and normal cells as well as lower toxicity in animal models as compared to cisplatin. These results suggest that GC20 is superior to cisplatin and other metal-containing compounds in overcoming drug resistance, severe toxicity, and over-reactivity.

However, ideal drug candidates not only need excellent pharmacological activity but also need suitable pharmacokinetic properties. However, so far, no reports have been found in this area for GC20. Therefore, in this study, we first developed a sensitive and specific liquid chromatography tandem mass spectrometry (LC-MS/MS) to determine GC20 in rat plasma and tissue homogenate. This novel analysis strategy was then successfully applied to a pharmacokinetic study of GC20 in rats to elucidate the plasma drug concentration and tissue distribution in vivo.

## 2. Results and Discussion

### 2.1. Optimization of LC-MS/MS Conditions

**Figure 1:**
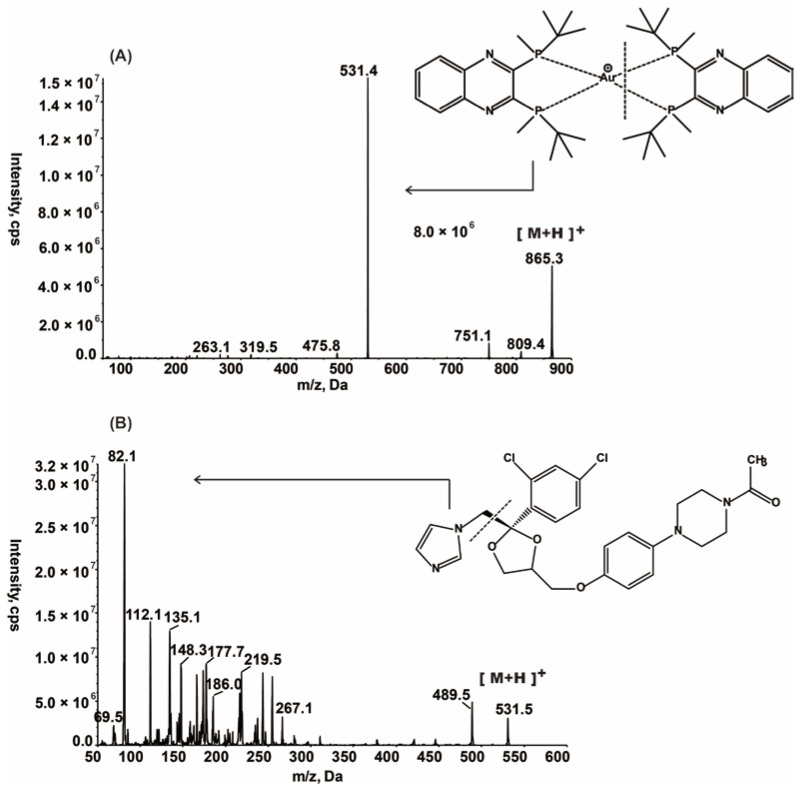
Product ion mass spectra of (**A**) GC20 (*m/z* 865.3→531.4), scan range 50–900 amu; (**B**) IS (Ketoconazole) (*m/z* 531.5→82.1), scan range 50–600 amu in the positive ionization mode.

First, a full-scan mass spectrum and a full-scan production spectrum of GC20 and IS were acquired in both positive and negative ion modes. Finally, a positive ionization mode was required for all due to a significantly superior ionization property in the positive ion mode. In the electrospray ionization (ESI) source, GC20 and IS predominantly form protonated molecules of [M + H]^+^. Figure 1 shows the product ion spectra of GC20 and IS. The major fragment ions were obtained at *m/z* 531.4 and 82.1 for GC20 and IS, respectively. Second, for chromatographic separation, three types of columns have been selected for study, including a Chrom Matrix HP amide column (5 μm, 3.0 × 100 mm I.D.) (Wuxi, Jiangsu, China), a Phenomenex Luna C18(2) 100A column (5 μm, 2.0 × 150 mm I.D.) (Los Angeles, CA, USA) and a Agilent Zorbax C18 column (3.5 μm, 2.1 × 50 mm I.D.) (Palo Alto, CA, USA). Finally, the Zorbax column was selected based on peak shape, retention time, and reproducibility. Third, erythromycin was first considered when selecting internal standards, because its mass-to-charge ratio was relatively similar to GC20. However, during the course of the experiment, erythromycin was found to be unstable in acidic conditions, and some acidic additives were added to the mobile phase because of the positive ion MS mode. This resulted in signal instability during the long-term detection of erythromycin. Ketoconazole was finally selected as an internal standard due to its good chromatographic behavior and stable signal response under the detection conditions of GC20.

Besides, in order to enhance the ion response and improve the peak shape, we have selected two types of acidic additives; one was ammonium acetate and acetic acid, the other was ammonium formate and formic acid. The results showed that although the former significantly improved the peak shape, excessively strong acid-induced ion suppression led to a decrease in the signal of the test compound, while the latter has a modest performance in improving peak shape and signal intensity. In addition, a gradient elution mode was optimized to achieve complete separation and nice peak shape in order to avoid interference between the analyte and the IS. The established gradient elution conditions have been confirmed to meet analytical requirements in terms of column equilibrium and carry over (see Supplementary Material). The chromatographic method established was stable and reproducible with almost no residual effect. Methanol was used as an organic phase to avoid tailing caused by acetonitrile. In order to reduce the contamination of the ion source, the first three minutes and the last one minute of the gradient elution were switched to waste.

### 2.2. Method Validation

#### 2.2.1. Specificity

**Figure 2:**
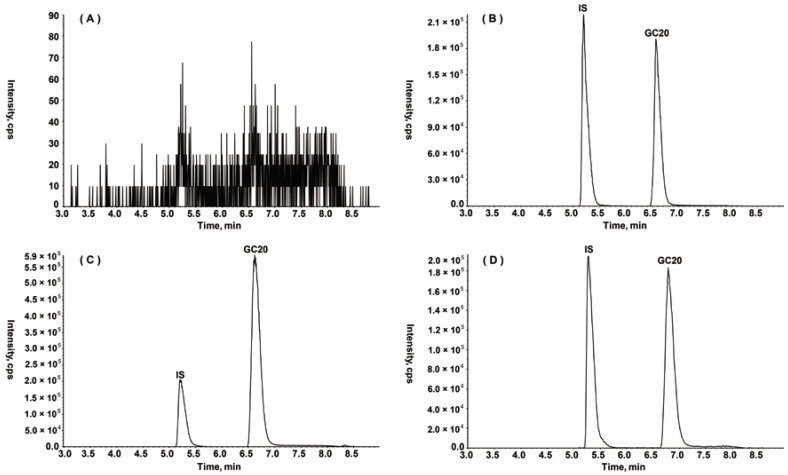
Representative chromatograms of GC20 and IS in rats: (**A**) blank plasma; (**B**) blank plasma spiked with GC20 at 500 ng/mL and IS; (**C**) plasma sample at 10 min after single intravenous administration of GC20 (1 mg/kg); (**D**) heart homogenate at 10 min after single intravenous administration of GC20 (2 mg/kg).

The chromatograms of the blank plasma, the blank plasma spiked with GC20 and IS, and the rat biological samples after intravenous injection (i.v.) GC20 to rats are shown in Figure 2. Under the established chromatographic conditions, no endogenous plasma components or other impurities interferences were observed at the peak region of GC20 (6.6 min) and IS (5.2 min). The peak shapes for the analyte and IS appeared to be symmetrical. These observations indicated that the specificity of the assessment was acceptable.

#### 2.2.2. Linearity and Sensitivity

The nine-point linear calibration curve exhibited good linearity over a concentration range of 1–1000 ng/mL for GC20 both in rat plasma and tissue homogenates. The correlation coefficient values were greater than 0.999 with a weighting factor of 1/x (Table 1). The LLOQ was 1 ng/mL, which was sufficient for the quantitative detection of GC20 in the pharmacokinetic studies and tissue distribution. At this LLOQ, signal noise ratio was greater than 10, and the accuracy and precision of plasma drug concentration detection were 2.1% and 8.9%, respectively.

#### 2.2.3. Precision and Accuracy

The intra-day and inter-day accuracy, which were expressed as the percentage error, were calculated by comparing the averaged measurements and the nominal values. The intra- and inter-day precisions were assessed by calculating the relative standard deviation. Accuracy and precision were assessed at three concentration levels (low/2 ng/mL, medium/50 ng/mL, high/1000 ng/mL). The test concentrations were selected according to US Food and Drug Administration guidelines for bio-analytical method validation (US Food and Drug Administration, 2001) [16]. The low concentration was within three times the lower limit of quantitation. The high concentration was near the upper limit of quantitation. The medium concentration was the intermediate value between low concentration and high concentration. As can be seen from Table 2, at all investigated concentration levels, the accuracy and precision data for GC20 in rat plasma were less than 7.5% and 7.4%, respectively. The results showed that this method was reproducible and reliable for the determination of GC20 in rats.

#### 2.2.4. Recovery and Matrix Effect

The mean recovery of GC20 in rat plasma at low, medium and high concentration levels ranged from 95.3–99.3% with an RSD of less than 5.5%, which indicated that the developed method was satisfactory for determining the plasma concentration of GC20. The matrix effect varied from 98.3–103.4% with an RSD of less than 4.8%, which indicated that no apparent ionization interference was found (Table 3).

#### 2.2.5. Stability Studies

The stability of GC20 under various storage conditions, including room temperature for 12 h, frozen at −80 °C for 7 days and 60 days, three freeze-thaw cycles and autosampler stability at 4 °C for 48h was evaluated at low, medium, and high concentrations (Table 4). All of the RE (%) and RSD (%) values were within ±10%, indicating that GC20 was stable under typical sample storage and processing conditions.

### 2.3. Pharmacokinetic Study in SD Rats

**Figure 3:**
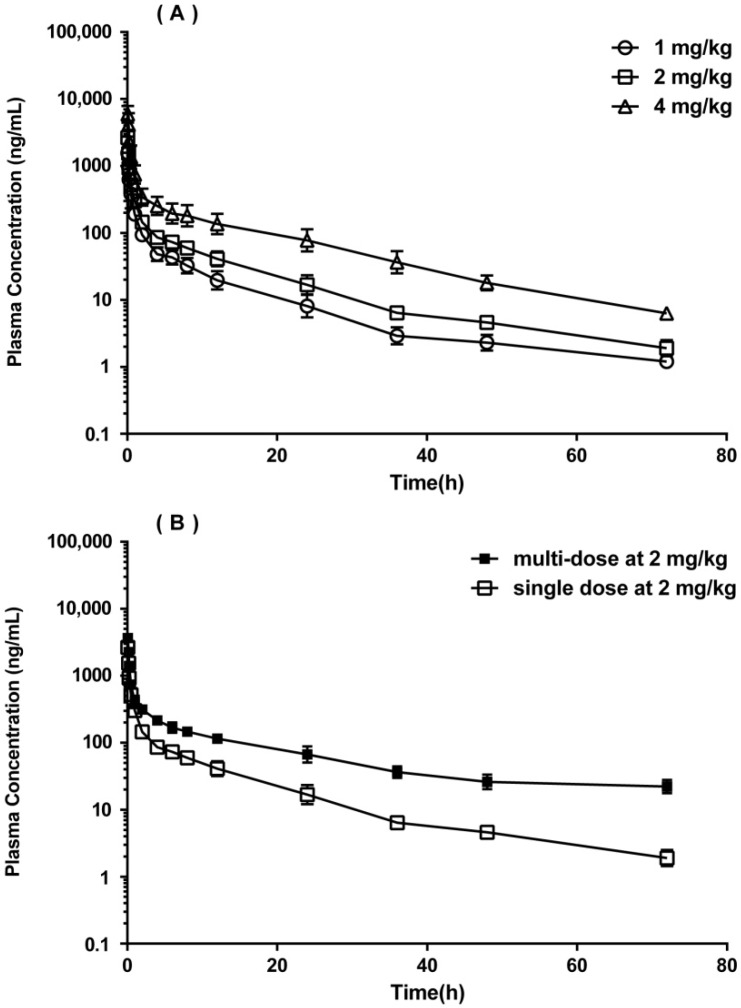
Mean plasma concentration-time profiles (semi-log graph) of GC20 in rats: (**A**) after intravenous administration of GC20 at three single doses (1, 2, 4 mg/kg); (**B**) after consecutive administration of GC20 for 7 days at 2 mg/kg compared to single administration of GC20 at 2 mg/kg. The data are expressed as mean ± SD from all rats in each dosing group (n = 6).

The validated LC-ESI-MS/MS method was successfully applied to a preclinical pharmacokinetic study of GC20 in rats after intravenous administration at a single dose and multiple doses. The mean plasma concentration-time profiles of GC20 after administration are shown in Figure 3. The pharmacokinetic parameters based on non-compartmental method are summarized in Table 5. Generally, GC20 showed dose-dependent plasma concentrations (C_5min_) and plasma exposures (AUC) after a single administration of low, medium, and high doses, and the elimination half-life of GC20 was about 11–13 h in rats. However, after consecutive administration of GC20 for 7 days, obvious differences in pharmacokinetic parameters were observed. A significant increase both in the plasma drug concentration and plasma drug exposure indicated that GC20 accumulated in rats after multiple doses. In summary, the linear kinetics of GC20 exhibited after a single administration would help predict plasma concentrations of GC20 in dose adjustments. The long elimination half-life of GC20 in vivo after a single administration indicated that GC20 would tend to accumulate during long-term use (experiments also have demonstrated this). The accumulation of GC20 may be due to saturation at elimination because the elimination half-life of GC20 in rats after multiple doses was twice that after a single administration.

### 2.4. Tissue Distribution

**Figure 4:**
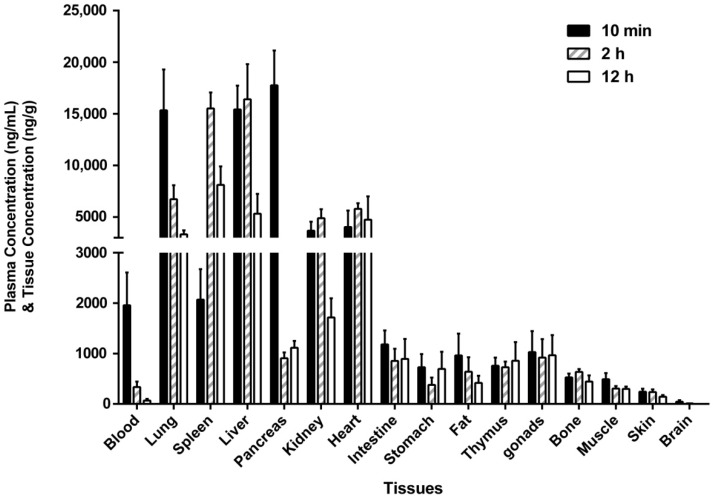
The distributed amount of GC20 in rat tissues at 10 min, 2 h, and 12 h after single intravenous administration of GC20 at 2 mg/kg (mean ± SD, n = 6).

After a single intravenous administration, GC20 was quickly and extensively distributed into various tissues (Figure 4). Among them, the highest concentrations of GC20 were found in the lung, spleen, liver, and pancreas. Then, the drug concentration in kidney and heart was also higher. Besides, there was a certain amount of drug distributed in the intestine, stomach, fat, thymus, gonds, bone, muscle, and skin. The concentration of GC20 was lowest in the brain, suggesting that it was difficult for GC20 to cross the blood–brain barrier. Moreover, the highest concentration of GC20 was detected at 2 h after intravenous administration in some tested tissues such as spleen, liver, kidney, and heart, indicating a delay in drug distribution in these tissues. In addition, the rate of metabolic clearance of GC20 in different tissues was also different. For example, in the lung, pancreas, and kidney, the elimination of GC20 was relatively fast, and the concentration of GC20 in these tissues had fallen below half of the corresponding maximum concentration at 12 h after administration. In the heart and thymus, the elimination of GC20 was very slow, and the tissue concentration was almost maintained at the same level within 12 h after administration. The tissue distribution results of GC20 could indicate in which organs GC20 may produce pharmacological effects or toxic side effects. For example, organs with the highest concentrations of GC20 in tissue distribution, such as the lung, liver, and pancreas, are highly likely to be the target organs for GC20 efficacy. On the other hand, the thymus as an immune organ not only has a certain amount drug distribution, but also the drug is slowly eliminated from the tissue, which is likely to cause drug accumulation and immunological side effects.

## 3. Material and Methods

### 3.1. Chemicals and Reagents

GC20 (purity > 99%) was supplied by the Department of Pharmacology, Shanghai Institute of Pharmaceutical Industry (Shanghai, China). Ketoconazole (internal standard, IS) was purchased from MedChemExpress (Monmouth Junction, NJ, USA). Methanol, formic acid, and ammonium formate were analytical grade and purchased from Sigma-Aldrich (St. Louis, MO, USA). Ultrapure water was prepared by Milli-Q Ultrapure water purification system (Millipore, Bedford, MA, USA).

### 3.2. Chromatographic and Mass Spectrometer Conditions

GC20 and IS were separated on a 50 mm × 2.10 mm, 3.5 μm ZORBAX 80A Extend-C18 column (Agilent, Santa Clara, CA, USA). The chromatography was performed at 40 °C, under gradient conditions. Gradient mobile phase system consisting of 0.1% formic acid added with 5 mM ammonium formate (A) and methanol (B) was applied at a flow rate of 0.3 mL/min. Retention times of GC20 and IS were 6.6 and 5.2 min, respectively. A typical injection of 5 μL was sufficient to obtain the required sensitivity. Run time of 10 min with a gradient elution: 0.0–1.0 min (10% B), 1.0–4.5 min (10–70%B), 4.5–6.5 min (70–95% B), 6.5–7.0 min (95–10% B), 7.0–10.0 min (10% B) were used.

The mass spectrometer was a Sciex API-4000 (Sciex, Redwood City, CA, USA) equipped with an electrospray ionization (ESI) source, operated in positive ionization mode, using multiple-reaction monitoring (MRM). The operating source conditions were optimized as follows: The ionSpray voltage was set at 5500 V, the turbo spray temperature at 500 °C, the ion source Gas 1 at 50 Arb, the ion source Gas 2 at 55 Arb, the curtain gas at 30 Arb and the Collision Gas at 10 Pa. The declustering potential and collision energies were 140 V and 40 eV for GC20, 100 V and 43 eV for IS, respectively. The entrance potential and collision cell exit potential were set to 10 V and 12 V for all the analytes. Detection of target ions [M + H]^+^ was at *m/z* 865.3 for GC20 and *m/z* 531.5 for ketoconazole (IS). The precursor-to-product ion transitions used were 865.3 → 531.4 and 531.5 → 82.1 for GC20 and IS, respectively. The data were acquired and evaluated using Analyst 1.5.1 (Sciex) software (Redwood, CA, USA).

### 3.3. Preparation of Standard Solutions and Calibration Samples

5 mg of GC20 was dissolved in 5 mL methanol to prepare stock solutions at a concentration of 1 mg/mL. The working solutions of GC20 in the desired concentration range were prepared by appropriate dilution of standard stock solution with methanol. All the solutions were stored at 2–8 °C and were brought to room temperature before use. The calibration standard (CS) samples for the rat plasma and rat tissue homogenates were prepared by spiking corresponding blank plasma or blank tissue homogenates with respective working solutions. The concentrations of CS samples in all biological matrix were 1, 2, 5, 10, 20, 50, 100, 200, 500, 1000 ng/mL.

### 3.4. Sample Preparation

The analyte of GC20 in rat biological samples was extracted using one-step protein precipitation. 50 μL of rat plasma or rat tissue homogenate was treated with 250 μL methanol containing IS (375 ng/mL). Vortex for 5 min to completely precipitate the proteins contained in the biological matrix. The mixture was then centrifuged at 18,000 rpm for 10 min (Sorvall Biofuge Stratos, Waltham, MA, USA), and 5 μL supernatant was injected for analysis.

### 3.5. Analysis Method Validation

To assess the specificity, blank plasma samples, plasma samples spiked with working solutions, and plasma samples after administration of drugs were detected. A ten-point linear calibration curve was constructed using a weighted (1/x) least squares linear regression by plotting the peak area ratios of analyte/IS versus nominated plasma concentrations over the range of 1.0–1000 ng/mL for GC20. The lower limit of quantitation (LLOQ), which was defined as the lowest concentration on the calibration curve, could fulfill the analytical requirement that S/N > 10 and the accuracy and precision were within ± 20% relative error (RE, %) and relative standard deviation (RSD, %), respectively. The linear curve concentration range from 1 ng/mL to 1000 ng/mL was determined based on the concentrations in vivo after GC20 administration and the mass spectrometric response of GC20. The LLOQ must ensure that the concentration of GC20 in rat plasma can be detected over a sufficiently long period of time (approximately 3–5 times the elimination half-life of GC20). However, the upper limit of quantitation (HLOQ) was limited by the saturation of the mass spectrometric signal of GC20, and the concentration above HLOQ exhibited a non-linear response.

Intra-day precision and accuracy were evaluated for six replicate samples at three concentration levels on the same day. Inter-day precision and accuracy were analyzed by the determination results in three consecutive days at three concentration levels. The accuracy was expressed as RE (%), and the precision as RSD (%). Both RE and RSD were expected to be within ±15% to be acceptable.

Extraction recovery was calculated by comparing the peak areas of extraction samples and post-extraction spiked samples. Matrix effect was evaluated by comparing the peak areas of post-extraction spiked samples and pure standard solutions. Post-extraction spiked samples were prepared as follows: Adding standard working solution into the mixed solution after protein precipitation of blank plasma to yield equivalent concentrations to corresponding extraction samples. Six replicates at three concentration levels were used for stability validation under a variety of storage and handling conditions. Samples were subjected to three freeze-thaw cycles, frozen at −80 °C for 7 days and 60 days, and storing at room temperature for 12 h. Post-preparative stability was evaluated by reanalyzing post-extraction samples kept in the autosampler at 4 °C for 48 h.

### 3.6. Pharmacokinetic Study in Rats

Twenty-four Spague-Dawley rats (twelve male and twelve female, 6–8 weeks old) were purchased from Shanghai SLAC Laboratory Animal Co., Ltd. (Shanghai, China). Animals were housed in an environment which controlled room temperature at 22 ± 2 °C and humidity at 55 ± 5%. Animals received 12/12 h light/dark cycle. Prior to the experiment, animals were fed a standard diet for one week to adapt to the laboratory conditions. Animal welfare and experimental procedures were strictly in accordance with the Guidelines of Animal Experiments of China Pharmaceutical University (Nanjing, China) and approved by the Animal Ethics Committee.

All rats were randomly divided into four groups, each group of six (three male and three female in each group). Three groups consisted of rats that received a single intravenous administration of GC20 at a single dose of 1, 2 or 4 mg/kg, while the fourth group consisted of rats that received a dose of 2 mg/kg on seven consecutive days. The doses for single administration was determined according to the pharmacodynamic doses and toxic doses of GC20 in animals reported in the literature [15]. The dose for multiple administrations was selected from the intermediate dose of a single administration. Dose solutions were prepared with 10% glucose solution. Before administration, rats fasted for 12 h. Blood samples (150 μL) were collected into tubes containing heparin (as an anticoagulant) via the ophthalmic veins at 0 min (i.e., before dosing), 5 min, 10 min, 15 min, 30 min, 1 h, 2 h, 4 h, 6 h, 8 h, 12 h, 24 h, 36 h, 48 h and 72 h. Then, the tubes were centrifuged at 8000 rpm for 5 min to separate the plasma. Plasma samples were then stored at −80 °C until further analysis. The pharmacokinetic parameters were calculated by Phoenix WinNonlin 6.0 (Pharsight, Mountain View, CA, USA).

### 3.7. Tissue Distribution in Rats

Eighteen SD rats (nine male and nine female, 6–8 weeks old) were purchased from Shanghai SLAC Laboratory Animal Co., Ltd. (Shanghai, China). After one week of adaptation, all rats were randomly divided into three groups, each group of six (three male and three female in each group). All rats were administrated GC20 solution at a single dose of 2 mg/kg vial the tail vein. The administered dose for tissue distribution was selected from the intermediate dose of single administration in the pharmacokinetic study. Blood samples were first collected via the ophthalmic veins and then rats were sacrificed to harvest tissues (i.e., heart, liver, spleen, lungs, kidneys, stomach, small intestine, brain, skin, muscle, fat, testes/ovaries, pancreas, thymus, and leg bones) promptly at 10 min, 2 h, or 12 h after dosing. To eliminate blood and other content, the tissues were thoroughly rinsed in water. Each tissue sample was homogenized into ten-fold volumes of water and then stored at −80 °C until further analysis.

## 4. Conclusions

First, a simple, sensitive and reliable LC-MS/MS method was developed and validated for the quantification of GC20 in rat plasma and rat tissue homogenate to assess the pharmacokinetic behavior and tissue distribution of GC20 in rats. Then, after intravenous administration, plasma levels of GC20 in rats were found to show a dose-dependent increase after single-dose administration. However, after multiple doses, GC20 accumulated in rats, and its elimination has become saturated. After a single administration, GC20 was widely distributed in various tissues and organs in rats. The highest levels were found in the lung, spleen, liver, and pancreas, while the lowest levels were found in the brain. This is the first report about the pharmacokinetic properties of GC20 in vivo. The results of pharmacokinetic studies contribute to a better understanding of the processes and mechanisms by which GC20 exerts therapeutic effects or side effects, as well as to assess the developmental prospects for GC20.

## Figures and Tables

**Table 1:** The linearity equations of GC20 in rat plasma and rat tissue homogenates over a concentration range of 1–1000 ng/mL.

Matrix	y = a + bx *		
	a	b	*r*
Plasma	3.92 × 10^−4^	2.34 × 10^−4^	0.9998
Heart	−1.04 × 10^−4^	2.23 × 10^−^^3^	0.9998
Liver	4.72 × 10^−4^	2.56 × 10^−^^3^	0.9997
Spleen	8.98 × 10^−4^	2.39 × 10^−^^3^	0.9998
Lung	−7.26 × 10^−4^	2.37 × 10^−^^3^	0.9995
Kidney	5.69 × 10^−4^	2.56 × 10^−^^3^	0.9996
Stomach	4.04 × 10^−4^	2.40 × 10^−^^3^	0.9999
Intestine	−9.33 × 10^−^^5^	2.55 × 10^−^^3^	0.9998
Pancreas	6.58 × 10^−^^3^	2.60 × 10^−^^3^	0.9998
Thymus	4.19 × 10^−4^	2.49 × 10^−^^3^	0.9998
Gonds	5.32 × 10^−4^	2.07 × 10^−^^3^	0.9999
Skin	1.06 × 10^−^^3^	3.12 × 10^−^^3^	0.9999
Muscle	8.77 × 10^−4^	3.61 × 10^−^^3^	0.9999
Fat	6.61 × 10^−4^	1.94 × 10^−^^3^	0.9997
Brain	6.85 × 10^−4^	2.14 × 10^−^^3^	0.9997
Bone	4.00 × 10^−4^	2.13 × 10^−^^3^	0.9997

* Linear equation with weight 1/x; *r* represents the correlation coefficient.

**Table 2:** The intra- and inter-day precision and accuracy of GC20 in rat plasma (n = 6).

Spiked Concentration (ng/mL)	Intra-day			Inter-day		
	Measured Concentration (ng/mL)	Precision (RSD%)	Accuracy (RE%)	Measured Concentration (ng/mL)	Precision (RSD%)	Accuracy (RE%)
2	2.0 ± 0.1	2.9	0.5	2.0 ± 0.0	2.2	0.2
50	50.8 ± 2.7	5.3	1.6	52.4 ± 0.8	1.5	4.8
1000	1020.9 ± 76.8	7.5	2.1	1074.3 ± 22.6	2.1	7.4

**Table 3:** Extraction recovery and matrix effect of GC20 in rat plasma (n = 6).

Analyte	Spiked Concentration (ng/mL)	Recovery (%)	Matrix Effect (%)
GC20	2	99.3 ± 5.5	100.0 ± 4.8
	50	97.2 ± 4.7	103.4 ± 2.5
	1000	95.5 ± 1.2	98.3 ± 1.3

**Table 4:** Stability of GC20 in rat plasma under different storage conditions (n = 6).

Storage Conditions	Spiked Concentration (ng/mL)	Measured Concentration (ng/mL)	Precision (RSD%)	Accuracy (RE%)
Room temperature for 12 h	2	1.8 ± 0.1	2.9	−9.2
	50	51.0 ± 0.4	0.8	2.0
	1000	1020.7 ± 23.6	2.3	2.1
Frozen (−80 °C) for 7 days	2	2.2 ± 0.2	1.8	8.2
	50	52.0 ± 0.9	1.7	4.1
	1000	1030.5 ± 15.9	1.5	3.1
Frozen (−80 °C) for 60 days	2	1.8 ± 0.1	2.8	−8.9
	50	47.3 ± 0.6	1.3	−5.4
	1000	970.1 ± 27.3	2.8	−3.0
Three freeze-thaw cycles	2	2.2 ± 0.1	1.5	8.7
	50	51.1 ± 0.3	0.7	2.3
	1000	1001.8 ± 19.0	1.9	0.2
Autosampler stability (48 h at 4 °C)	2	1.8 ± 0.0	2.6	−10.1
	50	45.2 ± 0.1	0.3	−9.5
	1000	901.3 ± 17.1	1.9	−9.9

**Table 5:** Pharmacokinetic parameters after intravenous administration (i.v.) of GC20 at a single dose (1, 2, 4 mg/kg) or after receiving a dose of 2 mg/kg on seven consecutive days. (mean ± SD from all rats in each dosing group, n = 6).

Non-Compartmental Parameters	Single Dose			Multiple Dose
	i.v. 1 mg/kg	i.v. 2 mg/kg	i.v. 4 mg/kg	i.v. 2 mg/kg
C_5min_ (ng/mL)	1564.6 ± 387.5	2657.6 ± 632.7	5902.6 ± 1955.2	3645.8 ± 388.3
AUC_0–t_ (h*ng/mL) *	1419.9 ± 345.5	2466.4 ± 298.3	7237.1 ± 2695.0	4551.2 ± 440.3 ^#^
AUC_0-∞_ (h*ng/mL)	1457.0 ± 354.5	2540.5 ± 308.6	7562.2 ± 2709.6	6936.7 ± 1005.4
t_1/2_ (h)	11.33 ± 0.57	11.24 ± 0.75	12.58 ± 1.80	25.1 ± 3.0
V/F (L/kg)	11.89 ± 3.35	13.00 ± 2.39	11.11 ± 5.28	10.6 ± 1.7
CL/F (L/h/kg)	0.72 ± 0.19	0.80 ± 0.10	0.60 ± 0.24	0.29 ± 0.04

*: AUC represents area under the plasma concentration versus time curve from 0–72 h. ^#^: AUC represents area under the plasma concentration versus time curve from 0–24 h. C_5min_: the plasma concentration of GC20 at 5 min after intraveous administration which could be directly obtained from the plasma concentration versus time curve.

## Supplementary Materials

The following are available online at https://www.mdpi.com/1420-3049/24/11/2082/s1.

## References

[1] Basenengquist K. (2006). Intraperitoneal Cisplatin and Paclitaxel in Ovarian Cancer. Obstet. Gynecol..

[2] Mok T.S., Wu Y.L., Thongprasert S., Yang C.H., Chu D.T., Saijo N., Sunpaweravong P., Han B., Margono B., Ichinose Y. (2009). Gefitinib or carboplatin-paclitaxel in pulmonary adenocarcinoma. N. Engl. J. Med..

[3] Kato H., Fukuchi M., Manda R., Nakajima M., Miyazaki T., Sohda M., Masuda N., Fukai Y., Tsukada K., Kuwano H. (2003). Efficacy and toxicity of nedaplatin and 5-FU with radiation treatment for advanced esophageal carcinomas. Anticancer Res..

[4] Li G., Chen X., Wu X., Xie J., Liang Y., Zhao X., Chen W., Fu L. (2012). Effect of Dicycloplatin, a Novel Platinum Chemotherapeutical Drug, on Inhibiting Cell Growth and Inducing Cell Apoptosis. PLoS ONE.

[5] André T., Boni C., Mounedjiboudiaf L., Navarro M., Tabernero J., Hickish T., Topham C., Zaninelli M., Clingan P., Bridgewater J. (2004). Oxaliplatin, fluorouracil, and leucovorin as adjuvant treatment for colon cancer. N. Engl. J. Med..

[6] Xie C.Y., Xu Y.P., Jin W., Lou L.G. (2012). Antitumor activity of lobaplatin alone or in combination with antitubulin agents in non-small-cell lung cancer. Anti-Cancer Drug.

[7] Giaccone G. (2000). Clinical Perspectives on Platinum Resistance. Drugs.

[8] Kelland L.R. (2000). Preclinical perspectives on platinum resistance. Drugs.

[9] Bruijnincx P.C., Sadler P.J. (2008). New trends for metal complexes with anticancer activity. Curr. Opin. Chem. Biol..

[10] Nobili S., Mini E., Landini I., Gabbiani C., Casini A., Messori L. (2010). Gold compounds as anticancer agents: Chemistry, cellular pharmacology, and preclinical studies. Med. Res. Rev..

[11] Gandin V., Fernandes A.P., Rigobello M.P., Dani B., Sorrentino F., Tisato F., Björnstedt M., Bindoli A., Sturaro A., Rella R. (2010). Cancer cell death induced by phosphine gold(I) compounds targeting thioredoxin reductase. Biochem. Pharmacol..

[12] Bernersprice S.J., Filipovska A. (2011). Gold compounds as therapeutic agents for human diseases. Metallomics.

[13] Lima J.C., Rodriguez L. (2011). Phosphine-gold(I) compounds as anticancer agents: General description and mechanisms of action. Anticancer Agents Med. Chem..

[14] Craig S., Gao L., Lee I., Gray T., Berdis A.J. (2012). Gold-containing indoles as anticancer agents that potentiate the cytotoxic effects of ionizing radiation. J. Med. Chem..

[15] Wang Y., Liu M., Cao R., Zhang W., Yin M., Xiao X., Liu Q., Huang N. (2013). A soluble bis-chelated gold(I) diphosphine compound with strong anticancer activity and low toxicity. J. Med. Chem..

[16] U.S. Department of Health and Human Services Food and Drug Administration (2001). Guidance for Industry, Bioanalytical Method Validation. Federal Register.

