# Supplementary material for: Pharmacokinetics and Tissue Distribution of a Novel Bis-Chelated Gold(I) Diphosphine Compound, Bis(2,3-bis(tert-butylmethylphosphino)Quinoxaline)Aurate(I), in Rats
Source: Molecules. 2019 May 31;24(11):2082. doi: 10.3390/molecules24112082 (PMC6600414; doi:10.3390/molecules24112082)
Supplement: Supplementary file 1 [file molecules-24-02082-s001.pdf]

## Supplementary material

### 1. Method validation of carry over

A calibration standard sample with a final GC20 concentration of 1000 ng/ml was prepared by spiking rat plasma with respective GC20 working solution. This sample concentration was corresponding to the upper limit of quantitation (HLOQ) of the calibration standard curve. This HLOQ sample was subjected to protein precipitation with five volumes of methanol containing IS (375 ng / mL). A blank control sample containing no GC20 and IS was prepared by selecting 50μL of rat blank plasma to be treated with 250 μL blank methanol. Estimate residue by detecting the blank sample immediately after detecting the HLOQ sample. The result (see supplement Figure 1) showed that no significant GC20 and IS were detected in the blank sample, indicating that the established chromatographic conditions in this manuscript has no residual effect.

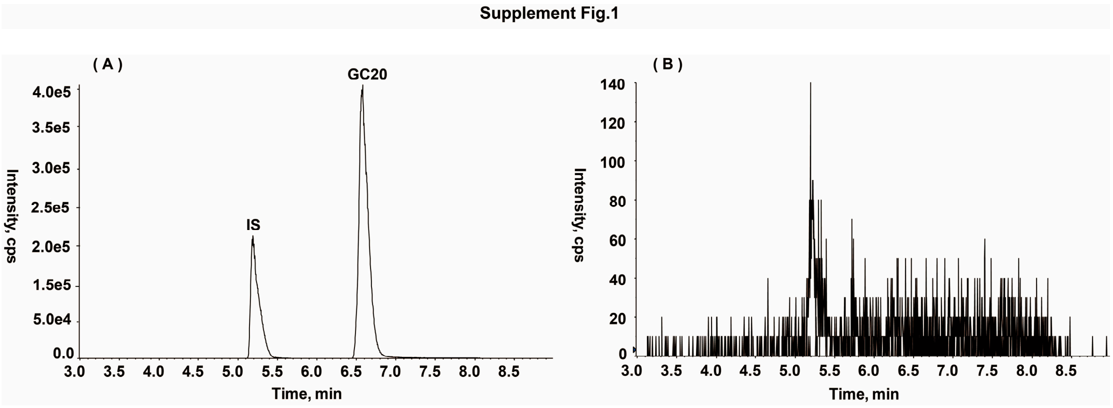


**Supplement Figure 1** Representative chromatograms of (A) blank rat plasma spiked with GC20 at 1000 ng/ml and IS; (B) blank rat plasma.

### 2. Method validation of column equilibration

Six quality control (QC) samples with a final GC20 concentration of 800 ng/ml were prepared by spiking rat plasma with respective GC20 working solutions. All QC samples were subjected to protein precipitation with five volumes of methanol containing IS (375 ng / mL) and the concentration of GC20 in these QC samples were detected continuously using the established chromatographic conditions. The detection chromatograms of the six QC samples were shown in supplement Figure 1. The mean retention time of GC20 in six QC samples was 6.57 ± 0.01 min and that of

IS was 5.19 ± 0.00 min. The results showed that the established gradient elution conditions had sufficient column equilibration time to ensure that the test results could be stably reproduced.

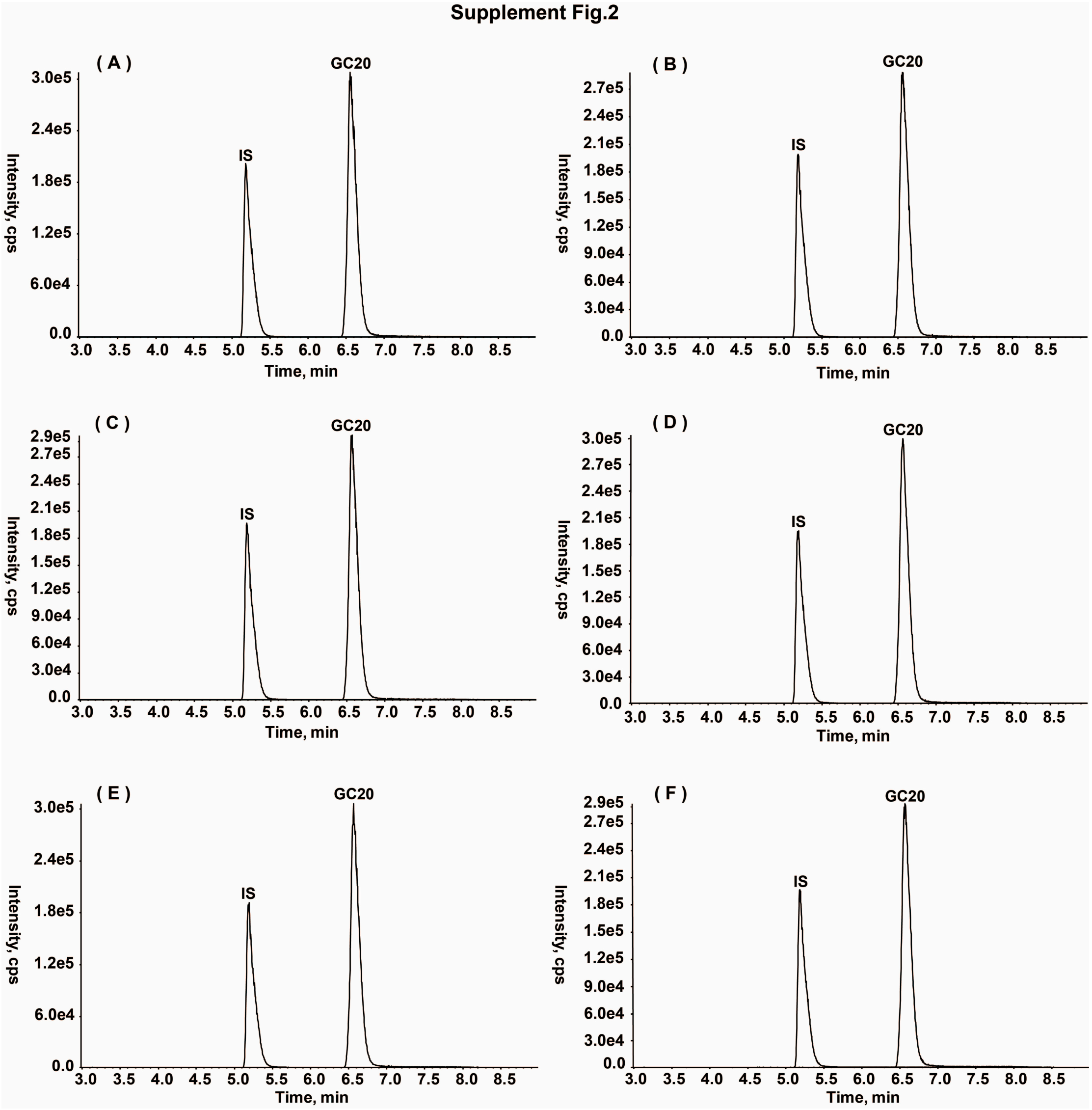


**Supplement Figure 2** Representative chromatograms of six quality control samples from (A) to (F). The samples were prepared by spiking rat blank plasma with GC20 at 800 ng/ml and IS.

## 3. Effect of 10% glucose solution on the LC-MS/MS profiles

Six male SD rats were selected and divided into two groups (three in each group). The rats in one group were intravenously injected with 10% glucose solution, and the rats in the other group were intravenously injected with saline. Blood samples were collected via the ophthalmic veins after administration and then centrifuged at 8000

rpm for 5 min to separate the plasma. The blank plasma from the group of 10% glucose solution was named as matrix A, while the blank plasma from the saline group was named as matrix B. Two types of calibration standard (CS) samples with a final GC20 concentration of 500 ng/ml were prepared by spiking the respective GC20 working solution into matrix A and matrix B, respectively. Three replicate samples were prepared for each type of CS sample. All CS samples were subjected to protein precipitation with five volumes of methanol containing IS (375 ng / mL), and then the analytes of GC20 and IS were then detected using established LC-MS/MS method. As seen from the supplement Figure 3 and supplement Table 1, GC20 and IS had no significant differences both in retention time and in signal intensity between the two types of CS samples. These results indicated that the dosing vehicle of 10% glucose solution for animal administration did not affect the determination of GC20 and IS in rat biological samples.

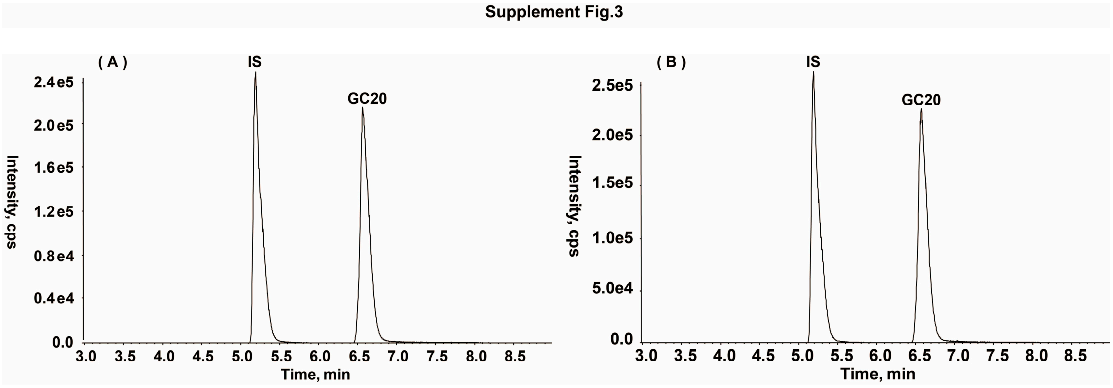


**Supplement Figure 3** Representative chromatograms of (A) calibration standard sample prepared by spiking GC20 at 500 ng/ml and IS into the blank plasma from the rat group of 10% glucose solution; (B) calibration standard sample prepared by spiking GC20 at 500 ng/ml and IS into the rat blank plasma from the saline group.

Supplement Table 1 The detection information in two types of CS samples (mean ±SD, n=3).

| analyte | parameter | CS sample of type A | CS sample of type B |
|---|---|---|---|
| | $t_R$ (min) | 6.58 ± 0.00 | 6.58 ± 0.00 |
| GC20 | Area (cps) | 1916001 ± 145399 | 1844448 ± 70481 |
| | Height (cps) | 229226 ± 14101 | 224361 ± 7332 |

| | | | |
|---|---|---|---|
| | $t_R$ (min) | 5.19 ± 0.01 | 5.19 ± 0.00 |
| IS | Area (cps) | 1899175 ± 47403 | 1836389 ± 11723 |
| | Height (cps) | 227856 ± 6531 | 248108 ± 1672 |

$t_R$ represent retention time.
Area represent peak area of the analyte.
Height represent peak height of the analyte.
CS sample of type A represent that the sample was prepared using the blank plasma from the rat group of 10% glucose solution.
CS sample of type B represent that the sample was prepared using the blank plasma from the rat group of saline.

56
